# Postprandial Effect of Orlistat on the Peaking of Lipid Level After Sequential High Fat Meals

**DOI:** 10.5812/ijem.2920

**Published:** 2012-04-20

**Authors:** Frederick S. Gabriel, Clarissa E. Samson, Zaynab R. Abejuela, Paula R. Sicat-Gabriel, Joan P. Sumpio, Manuel B. Zacarias, Leilani B. Mercado-Asis

**Affiliations:** 1 Section of Endocrinology and Metabolism, University Of Santo Tomas Hospital, Espana Blvd, Manila, Philippines; 2 Dietary Services, University Of Santo Tomas Hospital, Espana Blvd, Manila, Philippines; 3 Section of Cardiology, University Of Santo Tomas Hospital, Espana Blvd, Manila, Philippines

**Keywords:** Orlistat, Lipids, Postprandial Lipemia, Fatty Meal, Cardiovascular Disease

## Abstract

**Background:**

Postprandial lipemia has been found to be strongly associated with atherosclerosis due to its atherogenic and thrombogenic lipoprotein changes. This phenomenon occurs even in normal subjects especially after high fat meals. Orlistat, an anti- obesity drug, has been shown to address postprandial lipemia after a single high fat meal.

**Objectives:**

To compare the effects of orlistat and placebo on the postprandial lipid levels after sequential high-fat meals in healthy individuals with normal fasting lipid levels.

**Patients and Methods:**

Thirty-one healthy adult volunteers with normal fasting lipid levels were fed 50% fat meals (3 meals and 2 snacks of pre-weighted butter and bread). The subjects were blindly randomized to receive either placebo or orlistat 120 mg before each main meal. The outcome parameters were total cholesterol (TC), triglyceride (TG), high-density lipoprotein (HDL) cholesterol, low-density lipoprotein (LDL) cholesterol, and very-low–density lipoprotein (VLDL) cholesterol levels measured at fasting (0 h) and every 2 h thereafter, until the sixteenth hour. Additionally, we estimated the lipid levels at the fifth and ninth hour.

**Results:**

The non-orlistat group showed a significant postprandial rise in the levels of TG and VLDL, which began 4 h after breakfast (P < 0.05); this rise in levels was sustained until 9 h after breakfast for TG and up to 10 h after breakfast for VLDL. In contrast, only one significant rise in both TG and VLDL levels (at 4 h after breakfast) was noted in the orlistat group. The maximum mean difference from the baseline TG level for the orlistat group was lower than that for the non-orlistat group (0.22 mmol/L vs. 0.756 mmol/L, respectively). Similarly, the maximum mean difference from the baseline VLDL level from baseline in the orlistat group was only 0.099 mmol/L, which was lower than that in the non-orlistat group (0.588 mmol/L). LDL levels rose to a lesser extent in the orlistat group than in the non-orlistat group (0.268 vs. 0.362 mmol/L). The TC levels did not show a postprandial rise; instead, the levels reduced in both groups, with the orlistat group showing a higher reduction than the non-orlistat group (-0.288 vs. -0.188 mmol/L). The orlistat group did not show any significant differences in the HDL measurements.

**Conclusions:**

Administration of orlistat abolished the significantly sustained postprandial rise of TG and VLDL levels in healthy individuals who were fed sequential 50% fat meals.

## 1. Background

Dyslipidemia is an established risk factor for atherosclerosis, which leads to cardiovascular diseases. The National Cholesterol Education Program (NCEP) has developed a risk-factor assessment protocol on the basis of the measurements of fasting plasma lipid levels (1). However, even healthy individuals with normal fasting levels of lipids may show cardiovascular disease and related mortality. The Collaborative Atorvastatin Diabetes Study (CARDS) and the Justification for the Use of Statins in Primary Prevention: An Intervention Trial Evaluating Rosuvastatin (JUPITER) trials have documented this finding (2, 3). Although statins can prevent cardiovascular diseases, a large and growing number of coronary patients require therapeutic approaches other than mere low-density lipoprotein (LDL) cholesterol reduction (4, 5). One of the metabolic abnormalities in these patients is an exaggerated postprandial hyperlipemia. A number of clinical trials have shown that the level and duration of postprandial lipemia is directly related to the development of coronary heart disease (6).

So et al. (7) showed the occurrence of a significant lipid peak in healthy Filipinos 4–5 h after a single oral fat challenge test (OFCT) with a meal with 50% fat content. These findings are in congruence with the study conducted by Weiss et al. in 2008, which showed that postprandial lipemia tests with a 4-h interval between the meal and the measurement of lipid levels yielded highly reproducible results (8). Orlistat, also known as tetrahydrolipstatin, is a drug marketed primarily for treating obesity. Studies in healthy and obese human volunteers indicate that orlistat specifically reduces fat absorption from the diet by inhibiting triglyceride hydrolysis. (9) Therefore, about 30% of dietary triglycerides remain undigested and unabsorbed. In 2008, a local study by Abejuela et al. (10) showed that a single administration of orlistat after a single 50% OFCT could prevent the peaking of postprandial lipid levels.

Since most people consume about 3–5 meals every day, a considerable period of their lives are spent in the postprandial state. In effect, the body is exposed to more than 1 postprandial lipid peak in a day, thereby subjecting it to a greater degree and a longer period of hyperlipemia. Previous studies showed the effects of orlistat only after a single administration of the drug after a single high-fat meal.

## 2. Objectives

To date, there are no actual studies showing the effect/s of orlistat on the postprandial lipemia that occurs after successive meals. Hence, in this study, we aimed to compare the effects of orlistat and placebo on the postprandial lipid levels after sequential high-fat meals in healthy individuals with normal fasting lipid levels.

## 3. Patients and Methods

### 3.1. Study Subjects

We included healthy volunteers (male or female) with an age range of 18 to 45 years, who had normal body mass indices (BMI) (18.5–23.5 kg/m^2^) and normal fasting lipid levels, as mandated by the NCEP-Adult Treatment Panel III (ATP III) guidelines. We excluded individuals with clinical evidence of coronary artery disease, peripheral arterial disease, diabetes mellitus, hepatic and renal disease, and dyslipidemia (including family history of dyslipidemia). Additionally, we also excluded those subjects with concurrent acute or chronic medical conditions, subjects who were on maintenance medications, those who had donated blood within the past 3 months, subjects who were pregnant, and women who were menstruating at the time of subject selection. Comprehensive medical history-taking and physical examination were performed on all the selected volunteers. To ensure that the included subjects did not have any coexisting illnesses, we further screened them by performing complete blood counts; urinalysis; and assessments of fasting blood glucose, serum creatinine, and serum alanine transaminase (ALT) levels. In addition, we determined the fasting lipid profile (total cholesterol [TC], triglyceride [TG], high-density lipoprotein [HDL] cholesterol, LDL, and very-low–density lipoproteins [VLDL]) levels. Subjects with abnormal results were excluded from the study. Prior to enrolment in the study, subjects were informed of the nature of the study and the potential adverse events. Informed consent was obtained from each subject. The study was conducted in accordance with the ethical principles laid down in the Declaration of Helsinki.

### 3.2. Preparation of the High-Fat Meal

A high-fat meal consisting of 3 slices of bread and butter was prepared by a single dietician according to the subject’s daily caloric requirement. The subject’s desired body weight (DBW) was calculated from the subject’s height according to the Tannhausser’s Method:

DBW (kg) = [Height (cm) - 100] - 10% for Filipino stature.

The total daily caloric requirement (TCR) was then calculated by multiplying the subject’s DBW with his caloric requirement (cal/kg DBW/day), which was based on the subject’s level of physical activity. The TCR was subdivided into the following classes: 50% fat, 45% carbohydrate, and 5% protein. For every 5 g of fat load requirement, the subject was fed 1 teaspoonful of molten butter that was spread over 2–3 slices of bread. This was portioned into 3 meals (30% breakfast, 30% lunch, and 20% dinner) and 2 snacks (10% per snack).

### 3.3. High-Fat Meal and Orlistat Administration

The study subjects were blindly randomized (by drawing lots) to the orlistat 120 mg or placebo capsule group (dose, 1 capsule before each main meal). Subjects were admitted after 10 h of fasting. A heplock was inserted to facilitate serial venous blood extractions. The heplock was flushed with 1 cc of plain 0.9% saline solution between extractions, and the first 0.5 mL of blood was discarded prior to each specimen collection (2.5 cc per sample). The meals were provided as 3 main meals and 2 snacks, given at the following times: at 0700 (immediately after blood extraction for fasting lipid levels), 0900, 1200, 1500, and 1900. All participants were instructed to avoid any strenuous activity and to avoid drinking any liquid other than the water provided. Blood specimens were drawn at baseline/fasting (0 h) and every 2 h thereafter, until the sixteenth hour of observation. Additional lipid level determination was performed before and 4 h after the second meal (given at 1200) and at the fifth and ninth hour of observation. The lipid levels were measured by using a Hitachi 902 automatic analyzer by a single medical technologist who was blinded to the study. We provided diapers to the subjects in anticipation of possible adverse events such as oily spotting, flatus with discharge, and fecal urgency.

### 3.4. Data Analysis

We noted and analyzed the differences between baseline and postprandial TC, TG, HDL, LDL, and VLDL levels. The analysis of variance (ANOVA) was performed to analyze significant differences amongst the baseline and postprandial lipid measurements. We further analyzed the differences by using the Wilcoxon signed-rank test.

## 4. Results

Thirty-one healthy volunteers met the inclusion criteria and were subjected to the 50% OFCT. Sixteen subjects (51.6%) were randomized to receive orlistat (men, 9; women, 7), while 15 subjects (48.4%) received placebo (men, 8; women, 7). Both groups showed comparable baseline characteristics (Mann–Whitney U test) (Table 1).

**Table 1:** Baseline Profile of Healthy Volunteers

	Orlistat (n = 16)	Placebo (n = 15)	P value a
Sex, No. (%)			
Male	9 (56)	8	8 (53)
Female	7 (44)	7	7 (47)
Characteristic, Mean ± SD			
Age b	28.3 ± 7.3	22.1 ± 3.4	0.05
BMI, kg/m^2^ b, c	20.97 ± 1.53	20.95 ± 1.54	0.81
Actual weight, kg	53.69 ± 6.0	54.27 ± 7.04	0.81
Actual height, cm	160.9 ± 6.5	161.7 ± 7.1	0.75
Total calories/day	1,645.3 ± 176.5	1666.8 ± 190.7	0.75
Total fat/day	822.6 ± 88.2	833.4 ± 95.3	0.74
Fasting blood sugar, mmol/L	5.31 ± 0.50	5.39 ± 0.34	0.59
Cholesterol, mmol/L	4.32 ± 0.6	4.05 ± 0.49	7.33
Triglyceride, mmol/L	1.11 ± 0.39	0.92 ± 0.44	20.4
HDL, mmol/L	1.18 ± 0.29	1.2 ± 0.26	33.6
LDL, mmol/L	2.67 ± 0.56	2.43 ± 0.58	10.4
VLDL, mmol/L	0.50 ± 0.18	0.42 ± 0.20	8.88

^a^significant difference if P value is <0.05, Mann–Whitney U Test

^b^values from the Phil. Assoc. Of Study of Obesity and Overweight (PASOO)

^c^Normal range : 18.5–24.9

### 4.1. Effect on TC After 50% OFCT Challenge

**Figure 1:**
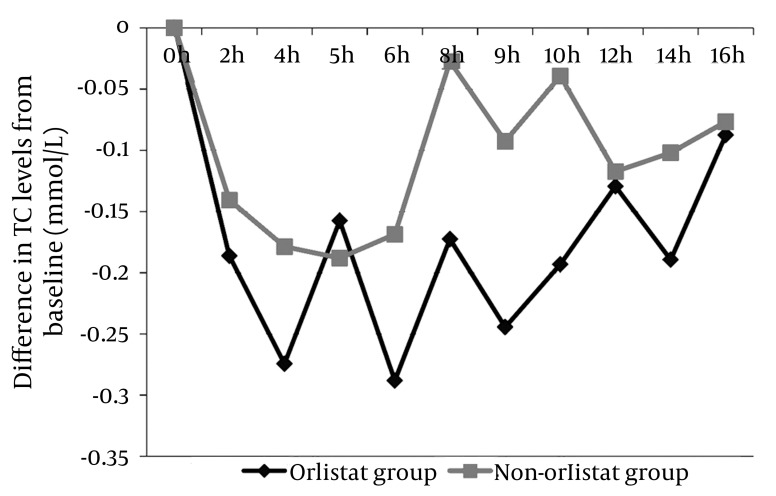
Differences in the Mean Total Cholesterol (TC) levels from the Fasting/Baseline Level Postprandial cholesterol levels were low in both the groups. However, a greater difference from the baseline levels was observed in the orlistat group (maximum mean difference from baseline, -0.288 vs -0.188 mmol/L).

The average postprandial TC measurements were below the baseline levels for both the orlistat and non-orlistat groups. However, the orlistat group showed a much lower postprandial TC level than the non-orlistat group (maximum mean difference from baseline, -0.288 vs -0.188 mmol/L) (Figure 1).

### 4.2. Effect of Orlistat on TG Levels After 50% OFCT Challenge

**Figure 2:**
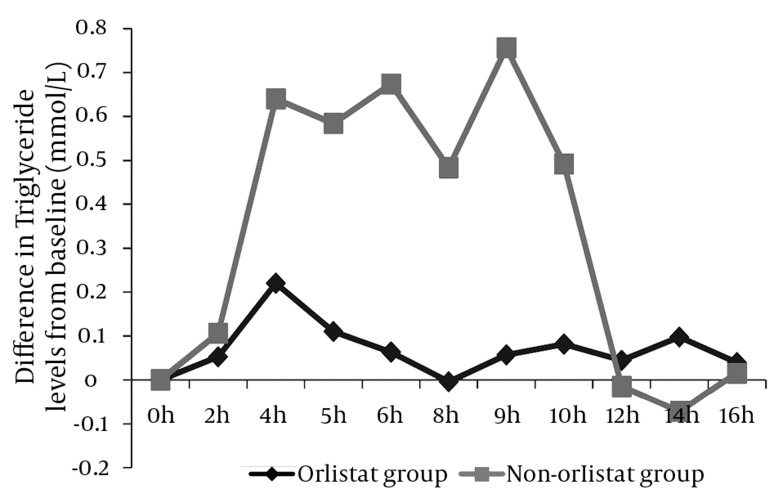
Differences in the Mean Triglyceride (TG) levels From the Fasting/Baseline Level No significant postprandial rise in TG levels was observed in the orlistat group. However, the non-orlistat group showed a significant postprandial rise 4 h after breakfast, which was sustained until the ninth hour post breakfast (P < 0.05).

In the non-orlistat group, a significant postprandial rise (P < 0.05) began 4 h after breakfast, and this increase was sustained until the ninth hour post breakfast (maximum mean difference from baseline, 0.756 mmol/L). In comparison, the orlistat group showed a minimal postprandial rise only after 4 h after each meal, with a maximum mean difference of 0.22 mmol/L from the baseline value. Apart from this rise, we did not observe any other postprandial rise in the orlistat group. (Figure 2).

### 4.3. Effect of Orlistat on the VLDL Fraction After 50% OFCT Challenge

**Figure 3:**
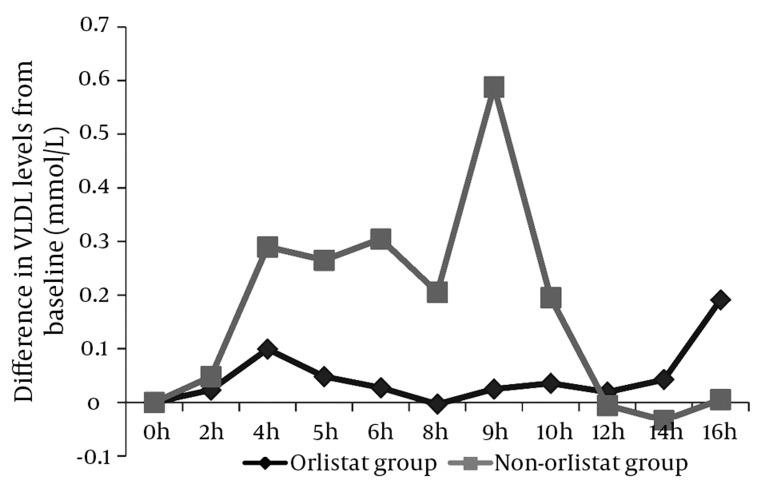
Differences in the Mean VLDL-Cholesterol levels From the fasting/ Baseline Level A minimal postprandial rise of only 0.099 mmol/L was observed in the orlistat group 4 h post breakfast; this rise was not sustained. The non-orlistat group, however, showed a significant postprandial rise 4 h after breakfast, and this rise was sustained until the ninth hour post breakfast (P < 0.05).

The findings for VLDL were similar to those for TG. In the non-orlistat group, a significant postprandial rise was observed starting at 4 h post breakfast and continued until the tenth hour after the meal (P < 0.05), with a maximum mean difference of 0.588 mmol/L from the baseline noted at the ninth hour post breakfast. In the orlistat group, we observed only a minimal postprandial rise of 0.099 mmol/L 4 h post breakfast. No other significant rise in postprandial levels was observed (Figure 3).

### 4.4. Effect of Orlistat on HDL Levels After 50% OFCT Challenge

**Figure 4:**
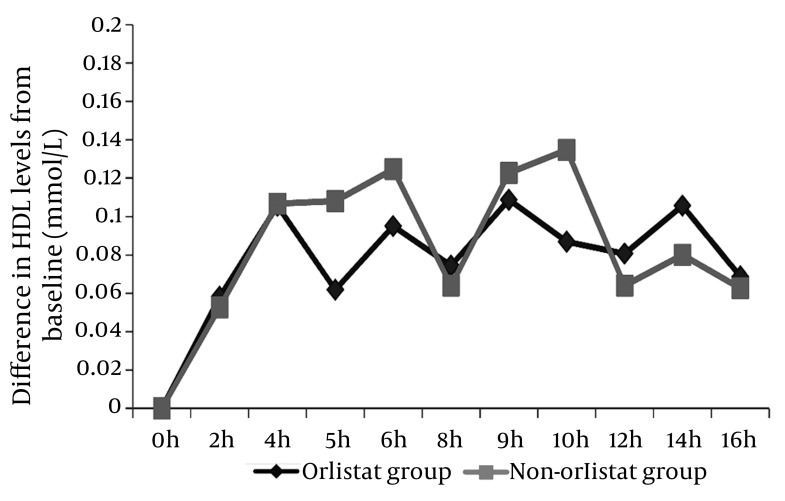
Differences in the Mean HDL levels From the Fasting/Baseline Level A significant difference was observed only for the measurements in the non-Orlistat group (P < 0.05).

Statistical analysis of the postprandial HDL measurements did not reveal significant differences in the orlistat group at any time point (P = 0.05); however, significant differences were noted in the postprandial HDL measurements obtained from the non-orlistat group (P < 0.05) (Figure 4).

### 4.5. Effect of Orlistat on the LDL Levels After 50% OFCT Challenge

**Figure 5:**
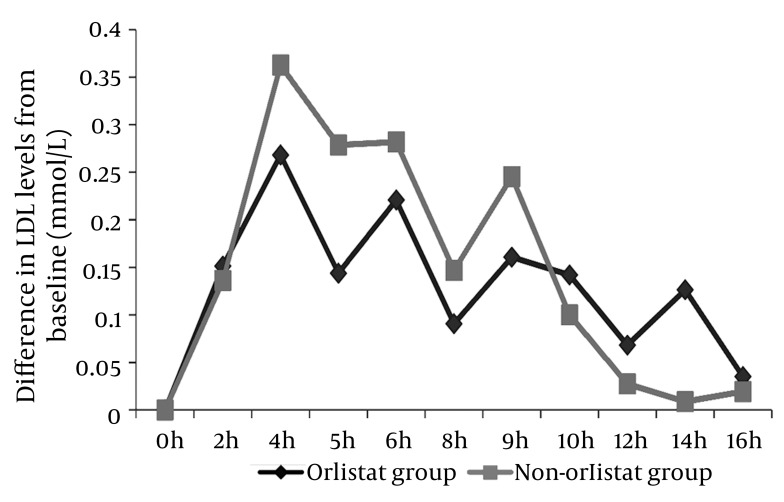
Differences in the Mean LDL From the Fasting/Baseline Level The orlistat group showed a lesser rise in postprandial LDL levels than the nonorlistat group, with maximum mean difference at 4 h after breakfast (0.268 vs. 0.362 mmol/L).

A significant postprandial rise was observed at the fourth and ninth hours post breakfast in both the orlistat and non-orlistat groups (P < 0.0001). However, the orlistat group showed a lesser increase in postprandial LDL levels than the non-orlistat group, with a maximum mean difference at fourth hour post breakfast (0.268 vs. 0.362 mmol/L) (Figure 5).

### 4.6. Adverse Effects of Orlistat

Five of the 16 (31.25%) subjects in the orlistat group reported adverse effects, which they described as mild and tolerable. The reported events were confined to the gastrointestinal tract; all events occurred within 30 h of orlistat intake. Of the 5 subjects, 3 had oily stools and 2 had oily spotting and flatus with discharge. All symptoms were self-limited and occurred only once. There were no drop-outs in the trial.

## 5. Discussion

Postprandial lipemia is a physiological phenomenon characterized by TG overproduction that occurs several times a day to cope with the nearly complete absorption after intake of dietary fat. The absorbed lipids are incorporated into chylomicrons to distribute dietary TG either for storage in the adipose tissue or for immediate use in metabolism. The dietary intake of fat frequently exceeds the actual need of the body. Under such conditions, the removal process of dietary TG may be ineffective, resulting in excessive postprandial lipemia that is linked to the generation of potentially atherogenic remnants of TG-rich lipoproteins (TRLs) (11). The direct effects are linked to the prolonged presence of these atherogenic lipoproteins in the plasma, which results in enhanced binding of these particles to the endothelium, thereby creating a marginated pool of endothelial cell-bound lipoproteins. The indirect effects are associated with the inflammatory aspects such as the activation of leukocytes and the endothelium or the activation of the complement system (12). Dietary lipotoxicity refers to the processes leading to end-organ damage following exposure to specific lipids (13). Endothelial cells may be particularly susceptible to the TRLs because of the significant level of lipoprotein processing that occurs via the interaction with hydrolytic lipases and constant exposure to plasma fatty acid and cholesterol (14).

Nordestgaard et al. could establish the direct correlation of non-fasting TG and the risk of myocardial infarction, ischemic heart disease, and death in 7,587 women and 6,394 men. In the same study, they also proved the direct correlation between TG and remnant lipoprotein levels (15). As a corollary to these results, Taguchi et al. showed that high serum levels of remnant lipoprotein cholesterol is a predictive hallmark for large-artery atherosclerosis in apparently healthy women (16). These studies suggest that non-fasting lipemia should be addressed even in apparently healthy individuals.

The anti-obesity effect of orlistat is achieved through inhibition of fat absorption. During a 4-year treatment period, statistical improvements were observed in fasting TC, HDL, and LDL levels (9). Several studies have also shown reduction of postprandial lipid peaks after the use of orlistat (17, 18). There are promising data on the effects of a single dose of orlistat in postprandial lipemia, but these do not allow us to draw firm conclusions regarding its effects on the lipoprotein subclass pattern in the real-life scenarios, wherein the meals are sequential. In a study by Abejuela et al., orlistat abolished postprandial peaking of TG and VLDL after a single high-fat meal (10). This study was performed in normolipemic subjects to assess the acute effects of orlistat on postprandial lipemia after intake of successive high-fat meals. The main finding of this study was that orlistat abolished the significantly sustained increase in TG and VLDL levels after ingestion of a standardized high-fat meal. Other factors such as diet and lifestyle may also contribute to postprandial lipemia. However, with the current lifestyle of Filipinos and the increasing incidence of insulin resistance, obesity, type 2 diabetes, and cardiovascular diseases, all of which may be linked to postprandial hyperlipidemia, a drug intervention should be considered as a preventive measure.

Our findings suggest that orlistat may be administered for primary prevention of atherogenesis and potential life-threatening cardiovascular disease, especially among patients in whom a high-fat diet is responsible for dyslipidemia. Our study also showed that the drug is well tolerated with minimal side effects. Our findings are consistent with the findings of Turker et al., who showed that orlistat plus a low-calorie diet were associated with a significant change from baseline to postprandial lipemia in normolipemic non-diabetic women (19). The limitation of this study is the relatively small number of volunteers who may not represent the entire Filipino population.

Administration of orlistat led to the abolishment of significantly sustained postprandial rise of TG and VLDL in healthy individuals who were fed sequential 50% fat meals. It has also reduced the extent of the postprandial increase of LDL, the lipid fraction that is considered atherogenic at high levels during fasting status. These findings may serve as a basis for the use of orlistat for primary prevention of cardiovascular diseases associated with postprandial lipemia.

## References

[1] (1993). Summary of the second report of the National Cholesterol Education Program (NCEP) Expert Panel on Detection, Evaluation, and Treatment of High Blood Cholesterol in Adults (Adult Treatment Panel II).. JAMA..

[2] Colhoun HM, Betteridge DJ, Durrington PN, Hitman GA, Neil HA, Livingstone SJ (2004). Primary prevention of cardiovascular disease with atorvastatin in type 2 diabetes in the Collaborative Atorvastatin Diabetes Study (CARDS): multicentre randomised placebo-controlled trial.. Lancet..

[3] Ridker PM, Danielson E, Fonseca FA, Genest J, Gotto AM, Jr., Kastelein JJ (2008). Rosuvastatin to prevent vascular events in men and women with elevated C-reactive protein.. N Engl J Med..

[4] Couillard C, Bergeron N, Prud'homme D, Bergeron J, Tremblay A, Bouchard C (1998). Postprandial triglyceride response in visceral obesity in men.. Diabetes..

[5] Mekki N, Christofilis MA, Charbonnier M, Atlan-Gepner C, Defoort C, Juhel C (1999). Influence of obesity and body fat distribution on postprandial lipemia and triglyceride-rich lipoproteins in adult women.. J Clin Endocrinol Metab..

[6] Hyson D, Rutledge JC, Berglund L (2003). Postprandial lipemia and cardiovascular disease.. Curr Atheroscler Rep..

[7] Sumpio JP, Mercado-Asis L, Zacarias M (2004). Postprandial lipid profile of healthy Filipino subjects after oral fat challenge test of varying fat contents.. Phil J Internal Medicine..

[8] Weiss EP, Fields DA, Mittendorfer B, Haverkort MA, Klein S (2008). Reproducibility of postprandial lipemia tests and validity of an abbreviated 4-hour test.. Metabolism..

[9] Litonjua A, Sy R (2004). Lipid Lowering Effect of Orlistat Beyond Weight Reduction in Overweight/Obese Filipino Patients: An RCT Study.. Phil J Internal Medicine..

[10] Abejuela ZR, Macaballug A, Sumpio J, Zacarias M, Mercado-Asis L (2009). Orlistat Abolishes Postprandial Lipid Peaking.. Int J Endocrinol Metab..

[11] Gardner DG, Dolores S (2007). Greenspan's Basic & Clinical Endocrinology..

[12] Karpe F (1999). Postprandial lipoprotein metabolism and atherosclerosis.. J Intern Med..

[13] Schaffer JE (2003). Lipotoxicity: when tissues overeat.. Curr Opin Lipidolol..

[14] Klop B, Proctor SD, Mamo JC, Botham KM, Castro Cabezas M (2012). Understanding postprandial inflammation and its relationship to lifestyle behaviour and metabolic diseases.. Int J Vasc Med..

[15] Nordestgaard BG, Benn M, Schnohr P, Tybjaerg-Hansen A (2007). Nonfasting triglycerides and risk of myocardial infarction, ischemic heart disease, and death in men and women.. JAMA..

[16] Taguchi M, Ishigami M, Nishida M, Moriyama T, Yamashita S, Yamamura T (2011). Remnant lipoprotein-cholesterol is a predictive biomarker for large artery atherosclerosis in apparently healthy women: usefulness as a parameter for annual health examinations.. Ann Clin Biochem..

[17] Zhi J, Melia AT, Guerciolini R, Chung J, Kinberg J, Hauptman JB (1994). Retrospective population-based analysis of the dose-response (fecal fat excretion) relationship of orlistat in normal and obese volunteers.. Clin Pharmacol Ther..

[18] Zilversmit DB (1979). Atherogenesis: a postprandial phenomenon.. Circulation..

[19] Turker I, Guvener Demirag N, Tanaci N, Uslu Tutar N, Kirbas I (2006). Effects of orlistat plus diet on postprandial lipemia and brachial artery reactivity in normolipidemic, obese women with normal glucose tolerance: A prospective, randomized, controlled Study.. Curr Therap Res..

